# Osteo Porosis

**Published:** 1891-08

**Authors:** A. Jasme

**Affiliations:** Savannah, Ga.


### COMMUNICATIONS.

### OSTEO POROSIS.

*To the Editor of* THE JOURNAL OF COMPARATIVE MEDICINE AND VETERINARY ARCHIVES.

Dear Sir :

I read with interest, an article by V. G. Hunt, in the July number of the Journal, on osteo porosis, which I understand as a criticism on an article written by me upon that subject. He states that the article is full of errors, and entirely misleading as to the pathology of the disease. As he has seen fit to expose my errors, of course, I am accorded the same privilege to show wherein he has erred.

In the first place, I think Mr. Hunt must not have read my article very closely, for he certainly did read it very misunderstandingly ; as he states that I claim the disease does not admit of successful treatment, and that, nevertheless, I admit that a quack friend of mine does accomplish a cure. Now, I think that in my article I made it plain to any person of ordinary powers of understanding that my article was simply a corroboration of the views of Dr. Bern, expressed by him, in an excellent paper published in the December number ; with a few exceptions, and the principal one was, that I disagreed with Dr. Bern as to the treatment, as he says that with our present knowledge of the disease, it would be folly to attempt to treat it ; while I do most certainly believe that it is amenable to treatment in certain stages of its course, and I do not think it would be folly to attempt it, for the same reason that I think it was not folly to attempt the treatment of smallpox a hundred years ago.

Now, as for my quack acquaintance, as Dr. Hunt chose to express it, is concerned, the gentleman referred to by me is a man of intelligence who has been a large stock raiser here for a number of years before this part of the country had the advantages of veterinarians, so was compelled for his own interest to overcome the disadvantage of losing valuable animals by the very prevalent disease, osteo porosis; and, by reading the best works at his disposal, together with experimenting for a number of years, he found a course of treatment which seemed to benefit, and I, for one, am always ready to take a suggestion which I consider will be of service to me in my profession, and at least give it a trial, whether it be from a quack or a negro stable hand. But, if Dr. Hunt will take trouble to inquire into the meaning of the word quack, he will find that it means a pretender, and the gentleman I refer to, does not pretend to be a specialist in treating "big head," nor does he affirm in anything that he cannot substantiate. I think we are all liable to commit errors, but before we should attempt to correct, we should be sure that we are in the right, and not jump at conclusions too quickly.

The gentleman asserts that, fifty years ago the disease was well understood, that every farmer could diagnose and in many cases so far cure it as to render the animal useful for a number of years. Now, if this be the case, it is strange to me that among our best authors of the present day no successful course of treatment is laid out.

He says that in certain parts of the West the disease is rare, and that the clod-hopping veterinarians of these sections find no trouble in *fixing them up*. If the doctor means by fixing up, that they cure or put into condition to be of service, I would be greatly obliged to him, if he will acquaint me with those *clod-hoppers*' modes of treatment, as I have great troubte to "fix up" many of the cases which I meet with.

Dr. Hunt says: But as the nature and courses of the ailment came to be understood, etc. . . . . Can I ask him to be kind enough to give us the nature and causes of osteo porosis. I would thank him very much for it, so would many of us who are and have been laboring for a number of years, and have as yet only arrived at suppositions which we consider as a first step towards the study of the *etiology* of osteo porosis.

It is supposed that a change of diet is to a certain degree a prophylactic measure; but, as the doctor agrees with me that the disease is more common in malarial regions, is not the decrease in number, in some parts, due to better drainage of our country, as is the case in malaria in the human family? and I consider from the fact of its malarial origin, that it is of infectious origin. I find a change of climate a great step toward the successful treatment of the disease; this again, I will advance as another reason for my theory of it being malarious.

The amenability to treatment that the doctor speaks of may be due to a lower type of infection brought about by better surrounding, as the country is being drained.

The statement, now, when they do occur are amenable to treatment—is, in my mind too extended. If I understand it correctly, when the fire is not too far advanced, *in well constructed* buildings ( improved dict ) we can extinguish it. If this is a fact, the greatest step towards the cure of osteo porosis is an early diagnosis. Easy to some, difficult to others : myself for one ; I acknowledge my incapability to differentiate osteo porosis in its early stage, from rheumatism, and any instruction on the subject will be gladly received.

Facts are certainly more or less wanting to prove the disease contagious, due to germs, etc.; but, are they less wanting to prove the contrary ?

Allow me to say a few words about a case that had been under my observation, and which I think proves the inefficiency of the diet, as prophylactic measure.

Mr. M., owns ten valuable horses, mares, colts—every one of which receives the very best of care and food : and twice a week bone phosphate preparation is given them in the feed, with the idea that it will strengthen the bony structures. Last November I was called to examine a colt one year old, and found him affected with osteo porosis. Now, to be short, I can find but one cause in this case : the stables are situated in a very malarial district.

In conclusion I would like to say a few words concerning the pathology of osteo porosis, but it is useless to me to take any more of your valuable space, and I can only refer to my article in June, 1891, and also to page 714, December, 1890, THE JOURNAL OF COMPARATIVE MEDICINE AND VETERINARY ARCHIVES ; in which Dr. Berns gives us, in my opinion, as much information concerning it, as any one who has written upon the subject.

The treatment of the disease is very uncertain, but in all *fixed up* cases I know of, the change of hygienic conditions, as removal to higher country early and thorough stimulation of the affected parts, good feeding, tonics, alteratives, a run at grass, in short, good attention have been of great advantage.

One opinion of Dr. Berns, I also wish to strengthen : The disease, I think, may be transmitted from sire or dam to offspring as I know of a stallion so affected; and the disease has developed in two of his colts (one died, the other developed lately). This one proof is not, however, sufficient to make it conclusive., but let us hope that the future will bring us more conclusive proofs as to the etiology of the disease, and that we will again hear, through the medium of your valuable Journal, of some practical facts that may be of service to us in the study of osteo porosis.

We want practical facts and not a series of comparisons, which are very spiritual indeed, but, take up too much space and valuable time.

Yours very respectfully,

A. JASME, V. S.,

Savannah, Ga.

